# Using SITAR (SuperImposition by Translation and Rotation) to estimate age at peak height velocity in Avon Longitudinal Study of Parents and Children

**DOI:** 10.12688/wellcomeopenres.14708.2

**Published:** 2018-12-14

**Authors:** Monika Frysz, Laura D. Howe, Jonathan H. Tobias, Lavinia Paternoster

**Affiliations:** 1 MRC Integrative Epidemiology Unit at the University of Bristol, University of Bristol, Bristol, UK; 2 Population Health Sciences, Bristol Medical School, University of Bristol, Bristol, UK; 3 Musculoskeletal Research Unit, University of Bristol, Bristol, UK

**Keywords:** ALSPAC, pubertal timing, growth, age at peak height velocity

## Abstract

Puberty is a time of substantial biological and psychological changes. One of the hallmarks of puberty is a rapid growth spurt, however its timing varies between individuals. The impact of pubertal timing on later health outcomes has been of interest in life course epidemiology, however its measurement can be challenging. Age at peak height velocity (aPHV) offers an objective measure of pubertal timing without having to rely on physical examination or self-report. We describe the derivation of aPHV estimates in Avon Longitudinal Study of Parents and Children (ALSPAC) offspring, using Superimposition by Translation And Rotation (SITAR) mixed effects growth curve analysis. ALSPAC is a rich source of phenotypic and genotypic data and given the importance of pubertal timing for later health outcomes, these data offer an opportunity to explore the determinants and consequences of aPHV.

## Introduction

Puberty is a period of significant biological and psychological changes in human development. One of its hallmarks includes a rapid growth spurt, the timing and speed of which varies between individuals, with marked sexual dimorphism.

The relationship of pubertal timing with adverse health outcomes in later life has been investigated previously ^1^. For example, previous studies reported an association between late menarche and increased risk of osteoporosis ^2^, whereas early pubertal timing has been found to be related to higher risk of obesity and cardiovascular disease in both men and women ^3^. Thus, investigating the influences of pubertal timing and understanding its relationship with later health outcomes is of great public health importance. While clinical assessment remains the gold standard for the assessment of pubertal status, this is difficult to achieve in large-scale studies. Self-reported puberty measures lack reliability ^4, 5^, and may be unpopular with study participants, potentially leading to large amounts of missing data. Another measure, age at peak height velocity (aPHV) provides an objective and non-invasive assessment of pubertal status.

The Avon Longitudinal Study of Parents and Children (ALSPAC) is a longitudinal birth cohort, which was established in the 1990s in the South West of England ^6^. ALSPAC is a rich source of data, including phenotypic and genetic data collected for the mothers, fathers and children.

This paper describes the application of Superimposition by Translation And Rotation (SITAR) mixed effects growth curve model (described previously by Cole *et al*. ^7^) for analysis of height in puberty and estimating aPHV in ALSPAC offspring which resulted in a new dataset being generated. Previous studies have shown this to be a suitable method for estimating aPHV ^8^, even with fairly sparse data ^9^.

## Methods

ALSPAC recruited a total of 14,541 pregnant women with expected delivery date between 1 ^st^ April 1991 and 31 ^st^ December 1992. Of these pregnancies, 69 have no known birth outcome, and of these 14,472 pregnancies, 195 were twin, 3 were triplet and 1 was quadruplet accounting for 14,676 known foetuses. These pregnancies resulted in 14,062 live births of which 13,988 children were alive at 1 year of age ^6^.

In order to increase sample size additional recruitment took place when the children were on average 7 years old (Phase II) and again between age 8 – 18 years (Phase III) resulting in a total of 15,247 enrolled pregnancies, of which 14,701 were alive at 1 year of age. For more details regarding eligibility and recruitment please refer to Boyd *et al*. ^6^.

These children have been followed up from birth (those recruited during Phases II and III were followed up from the time they joined the study) and data collection included questionnaires and clinical assessments. For the purpose of estimating age at PHV, only height measurements obtained by trained fieldworkers during assessment clinics were used. These clinics encompassed ‘children in focus clinics’ (CIF) to which a random 10% subsample of children were invited between the ages of 2 and 5 years, and six assessment clinics in late childhood (between age 7 and 13 years) to which all enrolled children were invited, three further assessment clinics in adolescence (ages 13, 15 and 17 years) to which all enrolled participants were invited. These data were restricted to include height measurements collected when the children were between 5 to 20 years of age which resulted in a total of 61,290 height measurements available for 10,236 participants of whom 5,099 were female and 5,137 were male. To maximise accuracy of the estimate of aPHV, these data were further restricted to include individuals with at least one height measurement for the following time periods: 5 to <10 years, 10 to < 15 years and 15 to 20 years (representing the pre-, peri- and post-pubertal periods for the majority of children). A total of 46,246 height measurements for 5,707 individuals (3,019 females, 2,688 males) were available for SITAR analysis.

SITAR is a mixed effects shape-invariant growth curve model, consisting of a mean growth curve along with three transformations (size, tempo and velocity), used to describe how each individual differs from the mean curve. The three SITAR parameters are size, reflecting up/down shift from the mean curve; tempo, reflecting left/right shift (on the age scale) which corresponds to the relative timing of puberty based on aPHV, and velocity reflecting stretching/shrinking of the age scale and hence describing differences in the rate at which individuals pass through puberty. For a detailed description of the method please refer to Cole *et al*. ^7^.

**Figure 1.**
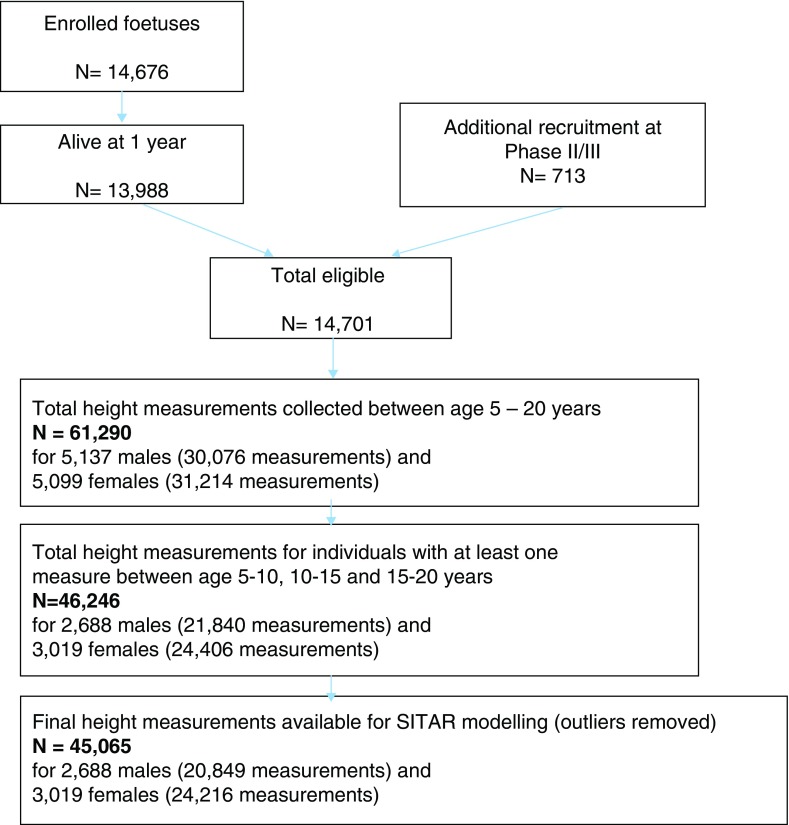
Avon Longitudinal Study of Parents and Children (ALSPAC) participant recruitment and height measurements available for analysis.

Following initial cleaning of height data (at least one height measurement available at each of the time periods: 5–<10 years, 10–<15 years and 15–20 years), as described above, the data were uploaded into R for further cleaning. Firstly, outliers with velocity exceeding 4 SDs in absolute value from the median for the dataset were removed (using velout and zapvelout functions; for more details see sitar package documentation in R ^10^), and initial SITAR model was subsequently fitted, for males and females separately, in R version 3.4.1. Secondly, standardized residuals exceeding 4 in absolute value were removed leaving a total of 45,065 height measurements for 5,707 individuals available for analysis with an average of 8 measurements (range 1–10 measurements) available per participant (see Figure 1 for details regarding participant recruitment). Of male and female participants in the final model, 32 (1.2%) and 18 (0.6%) had less than three height measurements, respectively.

### Dataset validation

**Figure 2.**
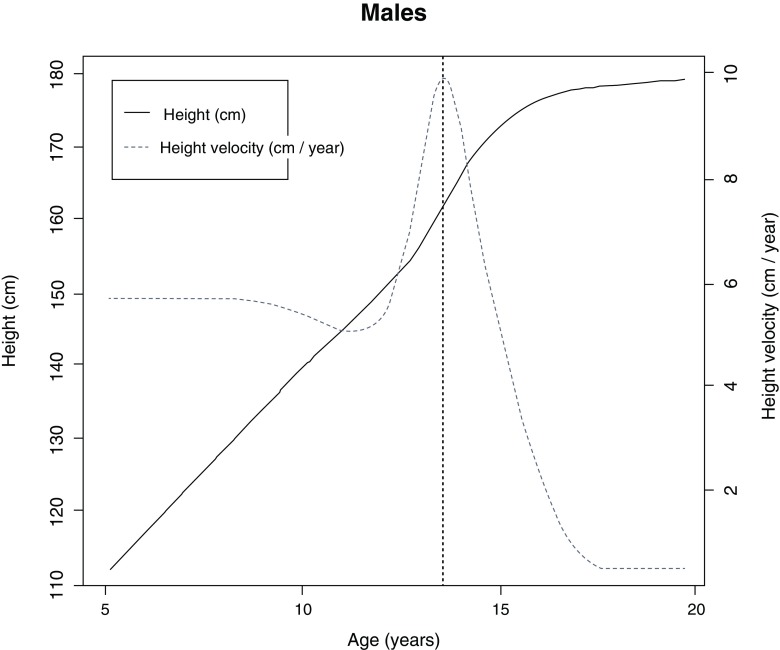
Mean growth curve (solid line) and velocity (dashed line) plots estimated by SuperImposition by Translation and Rotation (SITAR) for males. Vertical dotted line represents mean age at peak height velocity.

**Figure 3.**
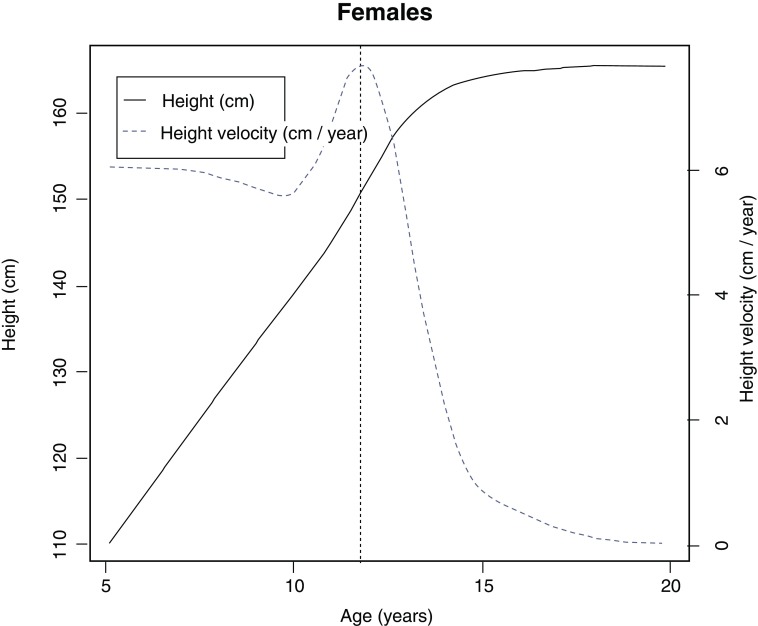
Mean growth curve (solid line) and velocity (dashed line) plots estimated by SuperImposition by Translation and Rotation (SITAR) for females. Vertical dotted line represents mean age at peak height velocity.

The final SITAR model was fitted with 5 degrees of freedom, for males and females separately, and it explained 98% and 98.3% of variance in the dataset for males and females, respectively. Mean aPHV (SD) was 13.6 (0.9) for males ( Figure 2) and 11.7 (0.8) for females ( Figure 3) and these estimates are similar to those reported in the literature ^11, 12^. The rich phenotype data available in ALSPAC provides unique opportunity to explore an extensive range of both determinants and consequences of aPHV; the aPHV variable could further be utilised as a confounder or mediator in a wide range of analyses. However, it needs to be noted that any findings may not generalise to non-white populations owing to >90% of sample being of White ethnic origin.

Table 1 shows mean and SD of additional variables that were estimated, which include: size (cm) (negative values indicating smaller whereas positive taller children and zero represents the mean), tempo (years) (negative values indicating early puberty, positive late puberty and zero represents the mean), velocity (years) (measure of intensity, positive values indicating short growth spurt, zero corresponding to mean velocity) along with peak velocity (cm) which measures the intensity of the pubertal growth spurt and aPHV measures its timing.

**Table 1.** Means and standard deviations of estimated SITAR growth parameters in Avon Longitudinal Study of Parents and Children (ALSPAC) offspring.

	Combined (N= 5,707)	Males (N=2,688)	Females (N=3019)
Variable name	Mean (SD)	Mean (SD)	Mean (SD)
aPHV (years)	12.6 (1.3)	13.6 (0.9)	11.7 (0.8)
Size (cm)	0.0 (6.3)	0.0 (6.5)	0.0 (6.0)
Tempo (years)	0.0 (0.9)	0.0 (0.9)	0.0 (0.9)
Velocity (cm/year)	0.0 (0.1)	0.0 (0.1)	0.0 (0.1)
Peak velocity (cm)	8.8 (1.5)	10.0 (1.1)	7.7 (0.8)

Abbreviations: aPHV (age at peak height velocity), SD (standard deviation)

## Ethical approval and consent

Ethical approval for the study was obtained from the ALSPAC Ethics and Law Committee and the Local Research Ethics Committees, full details of the approvals obtained are available from the study website ( http://www.bristol.ac.uk/alspac/researchers/research-ethics/).

Written informed consent was obtained from parents, and children were invited to give consent where appropriate. Study members have the right to withdraw their consent for elements of the study or from the study entirely at any time.

## Data availability

ALSPAC data access is through a system of managed open access. The steps below highlight how to apply for access to the data included in this data note and all other ALSPAC data. The dataset generated in this data note has been deposited within the ALSPAC data resource and is linked to ALSPAC project number B2325. Please quote this number to request required variables which have been described in this dataset (size, tempo, velocity, aPHV and peak velocity).

1. Please read the ALSPAC access policy (PDF, 627kB) which describes the process of accessing the data and samples in detail, and outlines the costs associated with doing so.
2. You may also find it useful to browse our fully searchable research proposals database, which lists all research projects that have been approved since April 2011.
3. Please submit your research proposal for consideration by the ALSPAC Executive Committee using the online process. You will receive a response within 10 working days to advise you whether your proposal has been approved.

If you have any questions about accessing data, please email alspac-data@bristol.ac.uk.

The ALSPAC data management plan describes in detail the policy regarding data sharing, which is through a system of managed open access.
