# Supplementary material for: Using SITAR (SuperImposition by Translation and Rotation) to estimate age at peak height velocity in Avon Longitudinal Study of Parents and Children
Source: Wellcome Open Res. 2018 Dec 14;3:90. Originally published 2018 Jul 25. [Version 2] doi: 10.12688/wellcomeopenres.14708.2 (PMC6171559; doi:10.12688/wellcomeopenres.14708.2)
Supplement: Peer review history

# Peer review: Referee response for version 1

**DOI:** 10.21956/wellcomeopenres.16018.r33563

**Published:** 2018-10-03

**Authors:** Hazel Inskip, Sarah Crozier

**Affiliations:** 1 MRC Lifecourse Epidemiology Unit, University of Southampton, Southampton, UK

Frysz and colleagues provide a valuable Data Note, describing the calculation of age at peak height velocity and associated parameters using the SITAR technique (reference 7 in the paper) in the ALSPAC cohort.  This technique reduces the complex information inherent in longitudinal measures of anthropometry to a smaller number of biologically-interpretable parameters.  The authors have clearly reported the methods used and we anticipate that the availability of these new growth parameters, alongside the information provided in this Data Note, will add significant value to the ALSPAC dataset as an epidemiological resource for the study of timing of puberty.

 We noted that the authors have removed outliers with velocity exceeding 4SDs which appears to be a reasonable method of dealing with clear measurement error.  However, it leads to questions as to whether there were any individuals with velocity below -4SDs, or size or tempo parameters below -4SDs or above +4SDs.  Why did the authors only remove those with velocity exceeding 4SDs?

 The analyses in this paper were restricted to participants with at least one height measurement in the time periods 5 to <10 years, 10 to <15 years and 15 to <20 years.  Could the authors describe why they chose these age brackets?  Also, the Methods of the paper could clarify that the assessment clinics in late childhood and adolescence are were not restricted to the 10% ‘children in focus’ sample; currently the description is somewhat ambiguous.

 Once the SITAR parameters of size, tempo and velocity have been calculated, two further variables have been created for each individual; ‘peak height velocity’ and ‘age at peak height velocity’.  It would be helpful if the authors could state specifically that these have been generated since age at peak height velocity is likely to be the output of greatest utility to future analysts using these data.  There is also potential confusion within the manuscript that ‘peak height velocity’ and ‘age at peak height velocity’ both sound rather similar to the SITAR ‘velocity’ parameter – the authors might clarify that they are separate variables.  In Table 1, the units for peak velocity should presumably be cm/year.  Also, it would be useful if all three of the axes in Figures 2 and 3 had units added.

 We noted two numerical discrepancies within the paper.  Firstly the text describes 14,541 pregnant women recruited to the ALSPAC study, whereas 14,676 enrolled foetuses are shown in Figure 1; this difference doesn’t appear to be attributable to multiple births.  Secondly, the numbers of male and female measurements available for SITAR modelling in Figure 1 do not add to the total number of measurements (i.e. 20,849 + 24,216 ≠ 40,037).

 The paper describes how 46,246 height measurements were available before considering potential measurement error.  Outliers were removed with absolute velocity exceeding 4SDs and after initial model fitting those with absolute standardised residuals exceeding 4SDs were also removed.  This left 40,037 height measurements available for SITAR modelling, a reduction of 13%.  Since 0.006% of a normal distribution lies below -4SDs or above 4SDs, this is a surprising reduction in number of measurements, even in the context of a significant amount measurement error; a comment on this would be helpful.

 The abstract ends with a helpful allusion to the opportunities afforded by these new measures available in the ALSPAC study.  It would enhance the paper if the end of the Methods section could similarly describe how these measures might be used in the future.  In particular the rich phenotype data available in ALSPAC provides the opportunity to consider an extensive range of both determinants and consequences of age at peak height velocity; the age at peak height velocity variable could further be utilised as a confounder or mediator in a wide range of analyses.

 In addition we note the following minor points of wording:

 The second line of the second column of page 3 would better read “between the ages of 2 and 5 years”.

 Similarly, the ninth line of the second column of page 3 might be better worded “To maximise accuracy of the estimate of aPHV,”.

We have read this submission. We believe that we have an appropriate level of expertise to confirm that it is of an acceptable scientific standard.

# Response

**Published:** 2018-12-10

**Authors:** Monika Frysz

**Affiliations:** University of Bristol, UK

We noted that the authors have removed outliers with velocity exceeding 4SDs which appears to be a reasonable method of dealing with clear measurement error.  However, it leads to questions as to whether there were any individuals with velocity below -4SDs, or size or tempo parameters below -4SDs or above +4SDs.  Why did the authors only remove those with velocity exceeding 4SDs?

*We thank the referee for their helpful comments.*

*Prior to final SITAR* *modelling* *the data were uploaded into R and examined for outliers. Both velocity outliers and standardized residuals exceeding 4 SDs in absolute value were removed (as previously described by Cole et al [1]) - this has now been clarified in the manuscript. Individuals with size or tempo parameters below -4SDs or above +4SDs were not excluded.*

 The analyses in this paper were restricted to participants with at least one height measurement in the time periods 5 to <10 years, 10 to <15 years and 15 to <20 years.  Could the authors describe why they chose these age brackets?  Also, the Methods of the paper could clarify that the assessment clinics in late childhood and adolescence are were not restricted to the 10% ‘children in focus’ sample; currently the description is somewhat ambiguous.

*The time periods 5 to <10 years, 10 to <15 years and 15 to <20 years were select to represent at least one pre-pubertal, one peri-pubertal, and one post-pubertal measurement for most children.*

*Thank you for your comment re ‘children in focus clinics’ sample– the assessment clinics in late childhood and adolescence were not restricted to the 10% ‘children in focus’ sample. The sentence has now been edited to add clarity:*

*These clinics encompassed ‘children in focus clinics’ (CIF) to which a random 10% subsample of children were invited between the ages of 2 and 5 years, and six assessment clinics in late childhood (between age 7 and 13 years) to which all enrolled children were invited, three further assessment clinics in adolescence (ages 13, 15 and 17 years) to which all enrolled participants were invited.*

  Once the SITAR parameters of size, tempo and velocity have been calculated, two further variables have been created for each individual; ‘peak height velocity’ and ‘age at peak height velocity’.  It would be helpful if the authors could state specifically that these have been generated since age at peak height velocity is likely to be the output of greatest utility to future analysts using these data.  There is also potential confusion within the manuscript that ‘peak height velocity’ and ‘age at peak height velocity’ both sound rather similar to the SITAR ‘velocity’ parameter – the authors might clarify that they are separate variables.  In Table 1, the units for peak velocity should presumably be cm/year.  Also, it would be useful if all three of the axes in Figures 2 and 3 had units added.

*Thank you for your comments. We have now updated the manuscript and edited the table and figures.*

 We noted two numerical discrepancies within the paper.  Firstly the text describes 14,541 pregnant women recruited to the ALSPAC study, whereas 14,676 enrolled foetuses are shown in Figure 1; this difference doesn’t appear to be attributable to multiple births.  Secondly, the numbers of male and female measurements available for SITAR modelling in Figure 1 do not add to the total number of measurements (i.e. 20,849 + 24,216 ≠ 40,037).

*Thank you for your comments. Of the 14,541 pregnant women recruited to the study, 69 had no known birth outcome and the remaining 14,472 pregnancies accounted for 14,676 foetuses (of which 195 were* *twin* *, 3 were* *triplet* *and 1 was quadruplet).*

*The total number of height measurements for both males and females has now been corrected in the main body of text and in the flow chart.*

 The paper describes how 46,246 height measurements were available before considering potential measurement error.  Outliers were removed with absolute velocity exceeding 4SDs and after initial model fitting those with absolute standardised residuals exceeding 4SDs were also removed.  This left 40,037 height measurements available for SITAR modelling, a reduction of 13%.  Since 0.006% of a normal distribution lies below -4SDs or above 4SDs, this is a surprising reduction in number of measurements, even in the context of a significant amount measurement error; a comment on this would be helpful.

*Thank you for your comments regarding the final number of heights available for SITAR modelling. We can confirm that the number of heights has now been corrected. The final sample available for SITAR modelling was 45,065 (not 40,037), a reduction of 2.6%.*

 The abstract ends with a helpful allusion to the opportunities afforded by these new measures available in the ALSPAC study.  It would enhance the paper if the end of the Methods section could similarly describe how these measures might be used in the future.  In particular the rich phenotype data available in ALSPAC provides the opportunity to consider an extensive range of both determinants and consequences of age at peak height velocity; the age at peak height velocity variable could further be utilised as a confounder or mediator in a wide range of analyses.

*Thank you for your useful comments regarding the opportunities afforded by a generation of pubertal measures in ALSPAC. We have edited the manuscript accordingly:*

*The rich phenotype data available in ALSPAC provides unique opportunity to explore an extensive range of both determinants and consequences of age at peak height velocity; the age at peak height velocity variable could further be utilised as a confounder or mediator in a wide range of analyses.*

 In addition we note the following minor points of wording:

 The second line of the second column of page 3 would better read “between the ages of 2 and 5 years”.

 Similarly, the ninth line of the second column of page 3 might be better worded “To maximise accuracy of the estimate of aPHV,”.

*Thank you for your comments. We confirm that the manuscript has been edited.*

**

 1.            Cole, T.J., et al., *Using Super-Imposition by Translation And Rotation (SITAR) to relate pubertal growth to bone health in later life: the Medical Research Council (MRC) National Survey of Health and Development.* 2016. **45**(4): p. 1125-1134.

# Peer review: Referee response for version 1

**DOI:** 10.21956/wellcomeopenres.16018.r33565

**Published:** 2018-08-16

**Authors:** John M. Pettifor, Shane A Norris

**Affiliations:** 1 MRC/Wits Developmental Pathways for Health Research Unit , Faculty of Health Sciences, University of the Witwatersrand, Johannesburg, South Africa; 2 MRC/Wits Developmental Pathways for Health Research Unit , Faculty of Health Sciences, University of the Witwatersrand, Johannesburg, South Africa

Frysz and colleagues from the University of Bristol describe the use of the statistical programme, Superimposition by Translation and Rotation  (SITAR), to determine the age of peak height velocity (aPHV) in a cohort of children participating in the Avon Longitudinal Study of Parents and Children (ALSPAC). The results they obtained for both boys and girls (13.6 years and 11.7 years respectively) are similar to those obtained by other researchers in well-resourced countries.

 SITAR, a recently developed modelling approach by Tim Cole ^1^, has been used increasingly in longitudinal studies to describe secular and population trends in childhood growth and pubertal development using skeletal development (Tanner RUS score) and aPHV in both poorly- and well-resourced communities ^2^^-^^4^. Its advantages are that it assesses each individual's curve against the mean curve and is able to determine the size (amplitude of change), tempo (the timing of the onset of change) and velocity (rate of change) of each individual curve in relation to the mean curve. It is also robust with missing data. Cole in a paper published this year assessed the interval required between height measurements in individual adolescent children to optimize the determination of aPHV ^5^. Cole concluded that height measurements need not be taken more frequently than biennially to obtain an accurate assessment of the average aPHV. 

 The reviewers have a few minor comments, which might help to provide further clarity on the participants included in the cohort. The authors describe the recruitment of participants from the ALSPAC cohort and the inclusion criteria for height measurements to be included into the database used for this particular aspect of the study. It is however unclear if black or Asian children were included in the analytical cohort sample. The reason for this comment is that data are available to suggest the age of pubertal development and aPHV in black children may differ from that of white children living in both well- and poorly-resourced countries ^3^^,^^6^. Thus their inclusion might have influenced the mean values of aPHV obtained and the subsequent comparisons with other cohorts. 

 The inclusion of an individual into the SITAR study required that at least one height measurement was available at each of three different time periods, 5-<10 years, 10-<15 years and 15-20 years. Yet in their analysis of the average number of measurements available per individual, the average is given as 8 with a range from 1-10. It is unclear how children with only one measurement were included based on the criteria for inclusion given above. It is possible that the authors meant that the participants included had at least one measurement in any of the age bands.

 In the second paragraph of the Methods section, line 3, Phase II is referred to as being between age 8 - 18 years. We believe this should be Phase III.

We have read this submission. We believe that we have an appropriate level of expertise to confirm that it is of an acceptable scientific standard.

## References

[1] ColeTJDonaldsonMDBen-ShlomoY: SITAR--a useful instrument for growth curve analysis. *Int J Epidemiol*.2010;39(6) : 10.1093/ije/dyq115 1558-66 10.1093/ije/dyq115 20647267PMC2992626

[2] ColeTJMoriH: Fifty years of child height and weight in Japan and South Korea: Contrasting secular trend patterns analyzed by SITAR. *Am J Hum Biol*.2018;30(1) : 10.1002/ajhb.23054 10.1002/ajhb.23054 28833849PMC5811819

[3] ColeTJRoushamEKHawleyNLCameronNNorrisSAPettiforJM: Ethnic and sex differences in skeletal maturation among the Birth to Twenty cohort in South Africa. *Arch Dis Child*.2015;100(2) : 10.1136/archdischild-2014-306399 138-43 10.1136/archdischild-2014-306399 25409981PMC4316919

[4] PrenticeADibbaBSawoYColeTJ: The effect of prepubertal calcium carbonate supplementation on the age of peak height velocity in Gambian adolescents. *Am J Clin Nutr*.2012;96(5) : 10.3945/ajcn.112.037481 1042-50 10.3945/ajcn.112.037481 22990031PMC3642996

[5] ColeTJ: Optimal design for longitudinal studies to estimate pubertal height growth in individuals. *Ann Hum Biol*.2018; 10.1080/03014460.2018.1453948 1-7 10.1080/03014460.2018.1453948 29669435PMC6191888

[6] RamnitzMSLodishMB: Racial disparities in pubertal development. *Semin Reprod Med*.2013;31(5) : 10.1055/s-0033-1348891 333-9 10.1055/s-0033-1348891 23934693

# Response

**Published:** 2018-12-10

**Authors:** Monika Frysz

**Affiliations:** University of Bristol, UK

The reviewers have a few minor comments, which might help to provide further clarity on the participants included in the cohort. The authors describe the recruitment of participants from the ALSPAC cohort and the inclusion criteria for height measurements to be included into the database used for this particular aspect of the study. It is however unclear if black or Asian children were included in the analytical cohort sample. The reason for this comment is that data are available to suggest the age of pubertal development and aPHV in black children may differ from that of white children living in both well- and poorly-resourced countries ^3^ ^,^ ^6^. Thus their inclusion might have influenced the mean values of aPHV obtained and the subsequent comparisons with other cohorts. 

*We thank the referee for their helpful comments regarding the inclusion of participants from black and Asian ethnic groups.*

*We can confirm that of 2,685 males included in the final sample 15 (0.6% of the sample) were black, 17 (0.6%) were Asian, 10 (0.4%) were from other ethnic groups and 198 (7.4%) had missing data on ethnicity.*

*Of 3,018 females included in the final sample, 17 (0.6%) were black, 16 (0.5%) were Asian, 23 (0.8%) were from other ethnic groups and 234 (7.8%) had missing data on ethnicity.*

*We agree, that previous literature reports earlier onset of puberty in non-whites. However, given the small proportion of individuals from ethnic minorities in this sample, this is unlikely to have influenced estimates reported in our manuscript.*

*A comment regarding generalisability has also been added to the manuscript:*

*However, it needs to be noted that any findings may not generalise to non-white populations owing to >90% of sample being of White ethnic origin.*

 The inclusion of an individual into the SITAR study required that at least one height measurement was available at each of three different time periods, 5-<10 years, 10-<15 years and 15-20 years. Yet in their analysis of the average number of measurements available per individual, the average is given as 8 with a range from 1-10. It is unclear how children with only one measurement were included based on the criteria for inclusion given above. It is possible that the authors meant that the participants included had at least one measurement in any of the age bands.

*The inclusion criteria described (at least one height measurement available at each of the time periods: 5-<10 years, 10-<15 years and 15-20 years) applied to a sample selected prior to SITAR modelling. These data were further cleaned, following the initial fitting of the model, and outliers with velocity and standardized residuals exceeding 4 were removed. As a result, individual height measurements were removed (rather than individual participants). Of males and females included in the final sample, 32 (1.2%) and 18 (0.6%) had less than 3 height measurements, respectively.*

 In the second paragraph of the Methods section, line 3, Phase II is referred to as being between age 8 - 18 years. We believe this should be Phase III.

*Thank you for your comment – the manuscript has now been edited.*
